# Supplementary material for: Hierarchical motor adaptations negotiate failures during force field learning
Source: PLoS Comput Biol. 2021 Apr 19;17(4):e1008481. doi: 10.1371/journal.pcbi.1008481 (PMC8084335; doi:10.1371/journal.pcbi.1008481)
Supplement: S1 Text — (DOCX) [file pcbi.1008481.s001.docx]

**Supporting information S1 text:**

# **Trajectory adaptation in Experiment-2**

The PSPF is a position-dependent force field in which the force perturbes hand movements of participants predominantly in the biggining of the reach and minimally near the target. The trajectory adaptation pattern in the PSPF (Figs S1A, S1B and 1C) was similar to that in the VDCF. The PSPF perturbed the participants’ hand trajectories considerably in the first adaptation and de-adaptation trials, but their hands could reach the target with the magnitude of TE being not significantly larger than the target size (1^st^ adaptation trial: t(14)=0.261 p=0.798; 1^st^ de-adaptation trial: t(14)=0.097 p=0.924). The LD showed a monotonic change through the adaptation and de-adaptation phases although some small TEs (but larger than target size) were observed around the 2^nd^-4^th^ adapation trials (Fig S1A). As the TEs occurred as a consequence of over-compensation for the perturbation, their subsequent reaches might be easily adusted and little affected by the TE-driven adaptation process. Largely, the PSPF behavior followed a typical adaptation pattern with no TE and a monotonic decrease in LD, as in the VDCF, which can be explained well by the internal model adaptation alone.

The CPVF is a force field that is a combination of a position-depdendent and a velocity-dependent force fields with the two force directions opposed to each other (Fig 1C). The velocity-dependent perturbation is predominantly effective over the first half of the reach such that the hand is pushed towards the right of the target (‘+’ direction). On the other hand, the position-dependent perturbation becomes stronger as the hand approaches the target, which can lead to a large TE to the left (‘-’ direction). This force field was utilized to confirm that another TE-inducing force field other than the LIPF could induce the new null trajectory as observed in the Experiment-1. As we expected, large TEs were observed in the first adaptation trial (73.2 ± 50.0 (mean ± s.d.) mm, t(13)=4.905, p=2.874×10^-4^) as well as the first de-adaptation trial (26.0 ± 22.8 mm, t(12)=5.211 p=2.178×10^-4^) (Figs S1C and S1D). Although some participants showed non-monotonic change of LD (see individual data in Fig S1D), the inter-participant variance of LD change was higher in CPVF compared to LIPF (Fig 2D) such that the non-motonocity was not visible in the averaged data. This was not unexpected given that complexity of the CPVF where the velocity and position dependent part of the field push the hand in opposite directions. On the other hand, and importantly, we again observed a curved null trajectory after the de-adaptation phase of CPVF, which was clearly different from the initial null trajectory (t(13)=3.386, p=0.0049; see Fig 3). The CPVF thus could reproduce the curved null trajectory observed also after LIPF, providing further support for the presence of the TE-driven adaptaion process.

The the flat models (internal model adapation only) could again not reproduce the CPVF behaviors (Figs S2A and S2B). The flat models inevitably produce a monotonic decrease in the LD in the initial trials and reduce it to zero after de-adaptation. In contrast, the hierarchical models (Fig S2C) could again explain the CPVF behavior. The simulation again showed the necessity of the TE-driven adapation process in addition to the internal model adaptation to explain behaviors in the presence of faliures, or TEs.
