# Supplementary figures and images for: Hierarchical motor adaptations negotiate failures during force field learning
Source: PLoS Comput Biol. 2021 Apr 19;17(4):e1008481. doi: 10.1371/journal.pcbi.1008481 (PMC8084335; doi:10.1371/journal.pcbi.1008481)

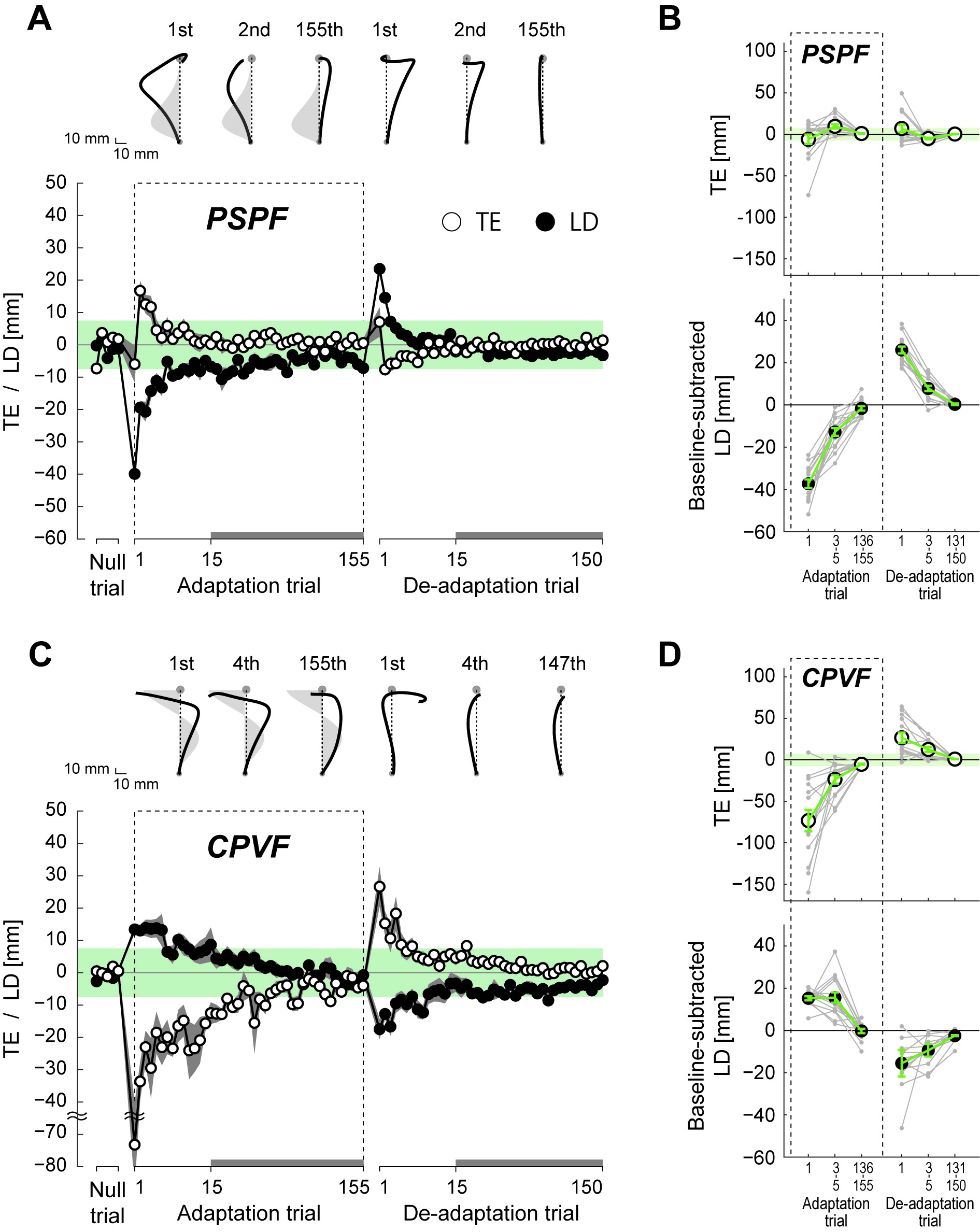

Supplement: S1 Fig — Trajectory adaptation in Experiment-2: (A, C) The hand trajectories of two representative participants and learning curves in PSPF (A) and CPVF (C) averaged across all participants. Note that the scales differ between x and y axes to clearly show trajectory changes along the x-axis. The light gray shades behind some trajectories represent a schematic image of the force field. The adaptation of the TE and LD are shown by traces with open circles and filled circles, respectively. The first 15 TE and LD values are plotted for every single trial, while the subsequent trials (indicated by thick gray lines at the bottom of the figure) are plotted for every five trials. The shaded gray areas around the lines represent standard errors. The light green zones represent the target width (radius: 7.5 mm). (B, D) The TEs and baseline-subtracted LDs in six trial epochs (1st, 3rd-5th, 136th-155th adaptation trials, and 1st, 3rd-5th, 131st-150th de-adaptation trials) in PSPF (B) and CPVF (D). Gray dots represent data from individual participants. The error bars indicate standard errors. The light green zone in the TE plots represents the target width. (TIF) [file pcbi.1008481.s003.tif]

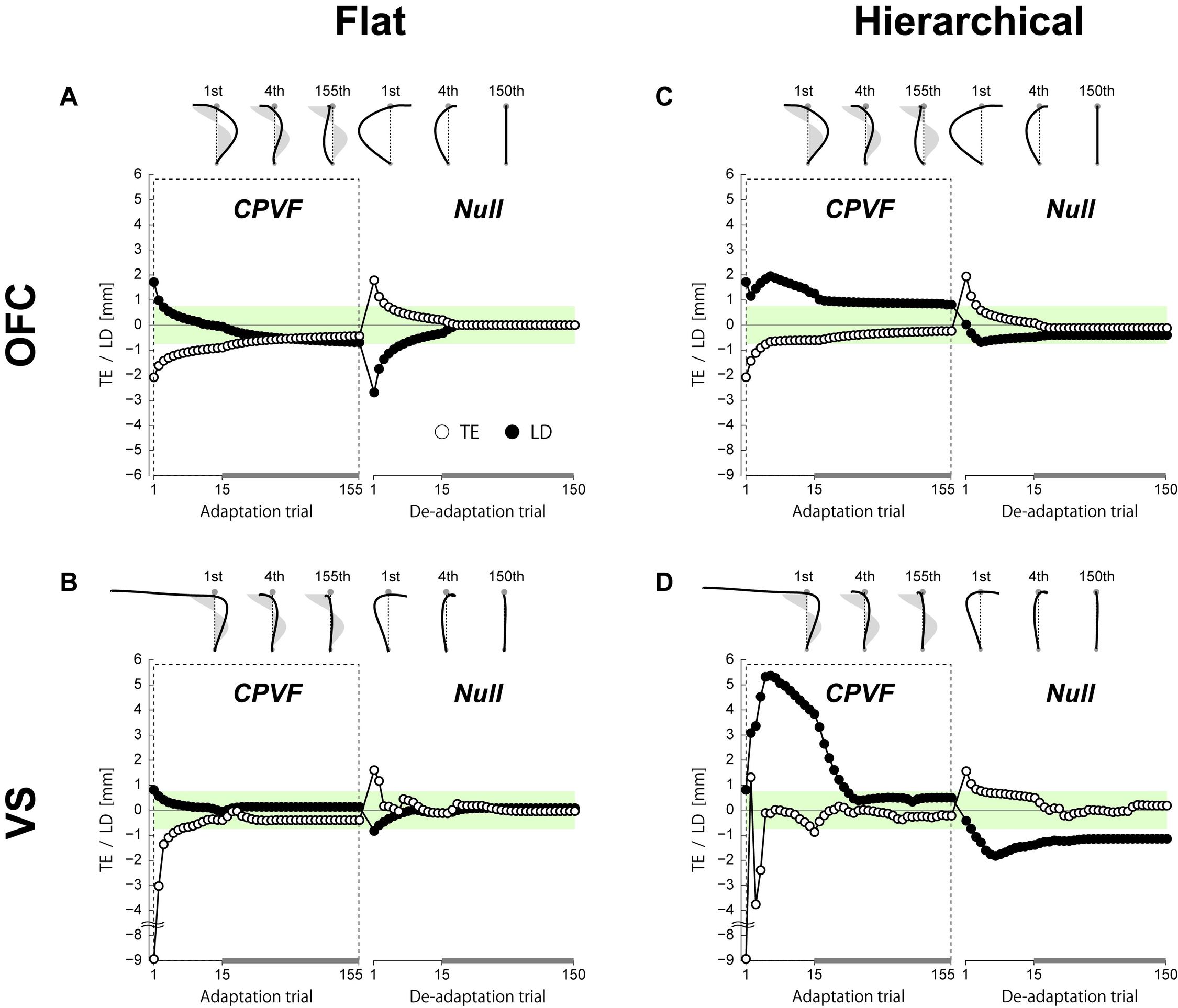

Supplement: S2 Fig — Simulation results for trajectory adaptation in CPVF, represented by TE (open circle) and LD (filled circle) by the flat (A, B)/hierarchical (C, D) OFC (upper panels) and VS models (lower panels). The flat learning models (only internal model adaptation) were unable to reproduce either the non-monotonic change in LD or the curved null trajectory with a persistent deviation after exposure to CPVF. However, the hierarchical OFC models (kinematic plan learning and internal model adaptation) successfully reproduced both. (TIF) [file pcbi.1008481.s004.tif]
