# Supplementary material for: Hierarchical motor adaptations negotiate failures during force field learning
Source: PLoS Comput Biol. 2021 Apr 19;17(4):e1008481. doi: 10.1371/journal.pcbi.1008481 (PMC8084335; doi:10.1371/journal.pcbi.1008481)
Supplement: Attachment — Submitted filename: PlosCompBiol_R1_Replies.docx [file pcbi.1008481.s005.docx]

We thank the editor and reviewers for their time and valuable comments which we think have improved the manuscript. We have revised the manuscript along the reviewers’ comments and suggestions. The changes have been indicated in red in the revised manuscript.

**Response to Reviewers**

**Reviewer #1**

*Major
My only major criticism is that the movement kinematics varied a lot across experiments and conditions, leading to the possibility that some aspects linked to online control also play a role. For instance, in Experiment 1, the magnitude of the position dependent FF produced larger deviations, and we do not know what would happen if the lateral deviations were comparable across force fields. The same concern holds for the control experiment #3, the virtual spring indeed reduces the target error but it also has an impact on many other kinematic parameters. Of course, it is very difficult if possible to only alter target error while matching all other kinematic parameters, but perhaps a complementary analysis such as subsampling trials with comparable kinematic errors to compare after effect would be useful, otherwise a note of caution seems necessary.*

Thank you for this suggestion. The LD in Experiment-1 were indeed larger in the VDCF condition (2.29[mean]±0.91[s.d.] cm) than the LIPF condition (1.02±0.47cm) (t(28) = 4.820, p = 4.539×10^-5^) in the 1^st^ de-adaptation trial although the peak velocity (t(28)=0.6625, p=0.513) was comparable. Therefore, following the reviewer’s suggestion, we conducted the subsampling analysis to verify our results. We sampled 6 participants from the VDCF condition who had the lowest LD in the 1^st^ de-adaptation trial, and 6 participants from the LIPF condition with the largest LD. The LD values were confirmed to be similar in distribution (t(10)=0.017, p=0.987). However, we still observed the same results as with the original data set; the new null trajectory appeared only in the LIPF condition (baseline-subtracted LD of the last 20 de-adaptation trials was higher than zero, t(5)=3.355, p = 0.020) but not the VDCF condition (t(5)=1.520, p=0.189).

Next, we checked the trajectories between the LIPF-Null and LIPF-PEC conditions of Experiment-3, and observed that the LD (t(29)=0.732, p=0.470) and peak velocity(t(29)=1.552, p=0.131) in the 1^st^ de-adaptation trial were comparable between the two. This suggests that the new null trajectory observed in the LIPF-Null condition cannot be explained by differences in the magnitude of LD or peak velocities at the beginning of the de-adaptation phase, and supports our conclusion that the presence of TE causes the new null trajectory. We have added this analysis to the result of Experment-3 on lines 276-279 (page 8).

However, we agree with the reviewer and recognize that there may still be other variables, that we cannot control in this study, and that may contribute to our observations. We have therefore added this point as a limitation of our study in the discussion on lines 507-523 (page 15).

*Specific:
Line 64: “motor movement” is a strange formulation.*

Thank you for this. We have removed “motor” from it on lines 80 (page 3).

*Intro, paragraph around line 75: The authors imply a strong link between explicit strategies, TE-mediated adaptation, and the fast state of motor adaptation which to my knowledge is not clearly established. One critical aspect is the impact of the paradigm, for instance the explicit strategy that counter target errors following visuomotor rotations to my knowledge does not directly apply to force field learning (perhaps it is a conclusion of this paper). Likewise, it is not clear to me that the implicit adaptation is only linked to the slow state. I would suggest to clarify the argument if I was missing anything, or that these concepts be presented with a bit more caution, keeping in mind that differences across adaptation paradigms (rotations and force fields) my hinder the correspondence between fast-slow, and explicit-implicit components.*

Although it is still few in number, an increasing number of studies seem to suggest that the ‘fast component’ of force field adaptation may also include an explicit component similar to vasomotor adaptations (Keisler and shadmehr 2010; McDougle et al., 2015; Schween et al., 2020). Our statement was made considering these studies. We have clarified this point further in the introduction on lines 96-99 (page 3).

*Related to the previous point, the possibility of rapid feedback adaptation (Crevecoeur et al., 7(1) ENEURO.0149-19.2019 1–16) is clearly linked to the fast state, but it is a SPE adaptation components in the authors’ terminology and likely linked to implicit adaptation as it produces systematic, one-trial lag after effect in a random scenario.*

Please note that the rapid adaptation in the Crevecoeur study is a within (single) trial change, while both our fast (TE-driven) and slow (SPE-driven) process are across trials, similar to, for example, the two-state model of Smith et al. (2006). However, we have discussed possible effects of adaptive control on the trial-to-trial trajectory adaptation observed in our experiments in the discussion on lines 520-523 (page 15).

*Lines 91-93: It is a bit of an overstatement that velocity dependent force fields have limited impact in end-point error. Often end-point corrections are not stable and it is not clear which error signal can be used in the case of non-zero terminal velocity or time out errors. Pls consider clarifying that although there is a clear difference in terms of TE magnitude, other aspects of behaviour, such as velocity and ability to stabilise, may also differ.*

We have modified the description of the VDCF on lines 109-112 (page 4) and have also discussed issues regarding other state variables as a limitation of our study on lines 507-523 (page 15).

*Lines 108-109: Although it is well defined in the Method section I would recommend giving more info about the force field at this stage (curl field? Orthogonal?)*

Thank you for this. We have further developed the force field descriptions in the introduction on line 131 (page 4) and the methods on lines 604-605 (page 17).

*I was under the impression that the hierarchical nature of the proposed model was not well motivated or not strongly supported in the data. It is fair to assume that a kinematic plan is selected prior to the derivation of the control law, and that the can some sequential organisation of kinematic (TE) and dynamic (SPE) adaptation. But this is potentially a property of the paradigm in which the target is fixed producing apparent hierarchy. However, there is evidence for online adaptation to changes in target location (Braun et al., J Neurosci, 29(20):6472– 6478), thus it is conceivable that such case the update of the kinematic plan be performed downstream of the selection of a control law based on sensory prediction errors. Is the hierarchy truly necessary, or can one imagine that the selection of the kinematic plan and the adaptation of the control law be processed in parallel?*

We would like to highlight three issues regarding the reviewer’s concern.

First, please note that we do not define hierarchy in terms of the temporal order of a process. But rather in terms of ‘process domination’- the process that regulates or determines another is considered to be hierarchically dominant to the other. That is, in our experiment target errors (TEs) led to two observations. a) the TEs drastically influence the trajectory changes across trials, which are usually monotonic when driven by internal model adaptation, and b) the TEs lead to a distinct and persistent null trajectory after de-adaptation. These results, in addition to our simulations, suggest that the internal model adaptation seems to be constrained (determined) by the dynamics of the TE driven process. We therefore consider the TE-driven process (which we call the kinematic plan adaptation) hierarchically dominant to the sensory prediction error driven (internal model) process.

Second, Braun et al. (2009) in fact shows that target jump (Experiment-2 in their study) provided with an unpredicted timing and low frequency (20%) do not lead to online adaptation (with a change in the control policy) but just online error correction (without any change in the control policy; see Fig 2 in their study). Thus, our results cannot be explained by the results observed during target jumps in the Braun study.

Finally, it is true that at the moment we cannot comment on the temporal question of whether the selection of the kinematic plan and the adaptation of control policy are processed one after the other or in parallel. Note that, as mentioned in the text, our purpose of simulating the results was just to emphasize the requirement of an additional TE driven process in the current motor adaptation models to explain our results. For this, we took the previous learning models (with their assumption of a discrete change in kinematic plan/parameters between trials) as it is. And even though these extended models, with their discrete changes in the kinematic plan, are able to explain our observations well, we cannot be certain that the part of the kinematic planning is not done online during the movement, rather than before it. This issue is beyond the scope of this study. We have now discussed this in the discussion on lines 518-523 (page 15).

*Again on the latter point: the apparent hierarchy is potentially induced by different timescales such that kinematic plan for a fixed target changes from trial to trial whereas adaptation based on sensory prediction errors uses continuous signals. However, in the Crevecoeur et al (cite above eNeuro) and in Braun et al (JNS, cited above), it is suggested that both sensory prediction error and kinematic plans can be updated within movement. Pls consider discussing the potential impact of the time-scales of adaptation to TE and SPE in the hierarchical organisation of the different adaptive components.*

As mentioned above, we have added this point in the discussion on lines 518-523 (page 15).

*Figure 3: the case strongly rests on the fact that long-lasting after effects in terms of lateral deviation characterise washout after position dependent force fields. It seems to be a robust and reproducible feature of the data and clearly the authors’ model can capture this aspect of behaviour, but is it fair to say that it is not clear why it happened in the first place? That it reveals differences between TE and SPE adaptation is fair, but I was under the impression that this finding was not clearly expected a priori.*

In addition to the non-monotonic trajectory changes, the long lasting (and robust) after-effects, observed after the LIPF in our pilot studies, was a key phenomenon that incited us to explore the target errors further. We then designed the experiment and collected the data to ensure that this phenomenon was indeed prevalent and reproducible, and the modelling helped us to explore the reasons behind both the phenomena.

*Please clarify the learning rule or update rule for alpha, was it fitted to the data? Is there a learning algorithm in the sense that the internal model is deduced, or is it just based on partial compensation of the force field with alpha lesser than one?*

Following the reviewer’s suggestion, we have added this point to the method on lines 814-815 (page 24). Alpha was determined from the observed data.

*Pls consider also sharing the simulation code to help the community playing with models of human adaptation and control.*

Definitely. We plan to share the code after the acceptance for publication.

**Reviewer #2**
*Major points

Lines 78-79: Miyamoto et al (2020) do not claim that the explicit and implicit components of adaptation occur “in parallel” but on the contrary, that they interact. In that sense, that citation would be more fitting several lines down (lines 80-83), where the authors mention work suggesting that task error and sensorimotor prediction error interact. This also applies at lines 475-476.*

Thank you for bringing this point up. As the work was not correctly cited, we have removed Miyamoto et al (2020) from the citation for the two-state model on lines 92-96 (page 3). Moreover, to more appropriately explain the relation between the two states of adaptation, we have changed “in parallel” to “in a non-hierarchical (flat) manner” on line 95 (page 4), lines 448-449 (page 13), and line 454 (page 13).

*Behavioral experiments: the LIPF group shows much larger TE errors at the end of the movement than the VDCF group showed LD errors (nearly 4 times more on average based on figure 1). This discrepancy is less pronounced in the PSPF versus CPVF comparison but still present (nearly 2 times more on average from figure S1). This consistent discrepancy may lead to increased feedback gains, or increased stiffness through co-contraction or postural control specifically for the TE groups to help reduce errors. This may explain baseline differences, as least in theory. For instance, a change in postural control may lead to a change in baseline trajectory to accommodate the biomechanics of the arm (Sergio & Scott, Exp Brain Res, 1998). An increase in feedback gainsmay may lead to changes in internal models as hypothesized in Miyamoto, Kawato, Setoyama and Suzuki, (1988, Neural Networks), and shown in humans (Maeda, Cluff, Gribble and Pruszynski, 2018, J Neurosci). The larger errors also suggest participants in these conditions experience stronger forces, which may lead to increased muscle fatigue and explain differences in the new baseline. Ideally the force-fields should be tuned so that the maximal lateral deviation at the distance of interest (LD for VDCF and TE for PIPF) is controlled and matching across conditions. Occasional full channel trials may also help rule out an effect of co-contraction as forces against a channel would signify net torques only. I leave to the authors to decide whether they would include such a control experiment, provide additional analyses to rule out alternative explanations, or explicitly mention this limitation where they deem appropriate in the paper.*

The reviewer has a valid concern. Like any study, we are unable to control all the variables between the force fields. This concern is also raised by Reviewer #1. In line with Reviewer #1’s suggestion, we have tested a subsample of the VDCF and LIPF participants to confirm that the differences in the LD and hand velocity magnitudes were not the cause of the observed differences (please see the reply to Reviewer 1). We have also confirmed that the LD and velocities were similar between the LIPF-Null and LIPF-PEC conditions.

On the other hand, we do not believe differences in muscle fatigue can explain the appearance of the new null trajectory in our TE inducing force-fields. This is because we observe new null trajectories in the LIPF-Null but not in the LIPF-PEC conditions, even though the participants train on the same LIPF before performing the de-adaptation phase in these conditions. We have added this point to the result on lines 294-298 (page 9).

However, we agree with the reviewer and recognize that there may still be other variables (like the ones the reviewer point out) that we cannot control in this study, and that may contribute to our observations. We have acknowledged this point as a limitation of our study in the discussion on lines 507-523 (page 15).

*Lines 275: “Half (15) of these participants had previously participated in Experiment-1.” Was analysis using this data corrected for multiple comparisons due to being used for several statistical tests? Also, if I understand this right, in line 294 “similar to Experiment-1” is a circular statement since half of the data comes from experiment 1. If the second half shows the same trend, it would be more informative to specify that.*

Thank for pointing out this point. We have used corrected p-values with Bonferroni multiple comparison for Experiment-3 and described this on lines 269-270 (page 8). Furthermore, we confirmed that the second half of participants shows the same results. We have added this results on lines 288-298 (pages 8-9).

*Modelling section: it seems unsurprising and therefore uninformative that a model with two “modules” (a task-error driven module and a sensorimotor-error driven module) can better account for the observed data than a model with only one module, because the data shows two uncorrelated behaviors. Indeed, the authors mention a similar point on lines 712-713. In that sense, the study in its current form supports the incompatibility of the data with a task-error blind (flat) model more than it supports the existence of a hierarchical model of the specific form presented here. Since the behavioral data already make the case for a task-error sensitive model, the current study would greatly benefit from a comparison between different plausible hierarchical models that include task error. The authors already lean in that direction in the discussion on the paragraph at lines 489-503, which was particularly interesting. Indeed, they mention the behavioral data of the current study is incompatible with Kim et al (2019)’s “Adaptation Modulation” model. What about the other two models that Kim and colleagues propose, namely the “Movement Reinforcement” and “Dual Error” models? It may be interesting to simulate each of them and assess their compatibility with the data presented here.*

We agree with the reviewer that two-module models can better account for the data than one-module models. However, the purpose of our modeling was just to emphasize the necessity of a hierarchical interaction with the well-studied internal model adaptation to explain the force field adaptation in the presence of TE or failure. As mentioned in the manuscript, we do not claim to propose new model as such. We believe that this comparison would help the reader to better understand the computational mechanism lacked by the previous (well accepted) models.

Moreover, please note that in the previous manuscript, we have already compared our models with other “two-module” models (specifically the two-state model and the Adaptation Modulation model) and explained why these models cannot explain our observations. This is also the case for both the “Dual Error” and “Movement Reinforcement” models.

The “Dual Error” model consists of a SPE-driven adaptation and a TE-driven adaptation both of which learn a visuo-motor rotation presented to participants in the study of Kim et al. (2019). Similar to the two-state model, the Dual Error model is a non-hierarchical model and the net adaptation of the participants is defined as the sum of the two. In the end, this model also considers only adaptation of the internal model (i.e. the exposed visuomotor rotation). In the case of our force field experiments, the Dual Error model can explain faster trajectory adaptations in the presence of TE but not the appearance of the new null trajectory. Like the Adaptation Modulation model, the TE-driven process of the Dual Error model determines a temporal feature of the SPE-driven adaptation (i.e., how fast arm trajectories adapts to the novel environment) but not a spatial feature (i.e., where the adapted trajectories converges) as in our models.

The “Movement Reinforcement” model modulates the retention of the SPE-driven internal model adaptation. The retention rate becomes higher and lower in the presence and absence of TE, respectively. Again, this model also considers only the adaptation of internal model and it thus can explain the temporal feature but not the spatial features of our observations.

We have added the discussion on the three models proposed by Kim et al. (2019) on lines 466-468 (page 13) and lines 473-481 (page 14).

*Lines 436 and 439: if the model decays very slowly, then it surely does not “converge” to a new baseline? Could the authors please clarify that point?*

We note that this line was unclear in the previous version. We have reworded this part to make it clearer on line 392 (page 11).

*Discussion: There is quite a lot of missing work of direct relevance to the current study, although I leave to the authors’ discretion what they deem interesting. The existence of a “new baseline” following reinforcement in visuomotor rotation tasks has been observed many times before (eg Galea et al. 2015 Nat Neuro, Schmuelof et al 2012 J Neurosci), though it remains partially unexplained (Holland et al 2019, J Neurosci), which makes this study potentially exciting. Possible causes that may be relevant are perceptual bias (Vindras et al 1998) and perceptual recalibration (Modchalingam et al 2019), with interesting parallel to the modelling part of this study. An exciting perspective of the current study is that one or both causes are sensitive to task error, which may also be worth pointing out. In line 501-503 the authors mention that the task error in the current study may be explicit, but explicitly reinforced reaching directions can still lead to an implicit new baseline (Holland et al 2019, J Neurosci), which may be worth clarifying. Finally, at lines 402-404, the study from Cashaback et al (2017, Plos Comp Biol) may be of interest to the authors, as it asks this question specifically.*

Thank you for suggesting the relevant studies. We have now clarified the difference between the new null (baseline) trajectory in our study and a persistent retention of learned movements reported by the previous studies (Shmuelof et al., 2012; Galea et al., 2015; Holland et al., 2019). These previous latter studies been reported the retention to occur after some reinforcement period where only binary success or failure feedback is provided. The retention is measured in the no feedback period following the movements, in which movement-related feedback is not available, and then when the feedback is available, a typical washout process appears with the movement quickly returning to the baseline level (Shmuelof et al., 2012). In contrast, in our study the new null trajectory persists for at least ⁓20 min of the de-adaptation period even when movement-feedback is available and without the reinforcement period. We have added this point to the discussion on lines 426-435 (pages 12-13).

Next, we considered the perceptual bias (Vindras et al., 1998; Ostry et al., 2010 JNS) and perceptual recalibration (Modchalingam et al., 2019) of the hand position as possible causes of the new null trajectories. Although they may partially contribute, again these effects alone cannot explain why the new null trajectories appear only in the TE-inducing but not the non-TE-inducing force fields. We have appended this point to the discussion on lines 415-419 (page 12).

Finally, we have discussed a possible effect of a binary (success or failure) feedback on trajectory adaptation. When the failure is indicated by a binary feedback but not a signed error feedback like TE, LD (SPE) may be more prioritized to compensate for as suggested by Cashaback et al. (2017). We have added this point on lines 546-549 (page 16).

*Minor points

Line 83: I find the term “parallel interaction” ambiguous, as “parallel” suggests independent processing, that is, it suggests no interaction. Some rewording may benefit the general reader by removing the ambiguity.*

We agree with the reviewer. As mentioned in the reviewer’s first ‘major point’, we have changed this statement to “in a non-hierarchical (flat) manner” on line 95 (page 4), lines 448-449 (page 13), and line 454 (page 13).

*Line 297: “PEC-LIPF” I assume the authors meant “LIPF-PEC”?*

Thank you for this. We have corrected it.

*Figure S1: I believe the legends (open and closed circle) is missing on this figure? I only find it mentioned in the caption.*
Thank you for this. We have added it.

*Line 610: How long was the hand constrained at the end?*

The constraint force is active from the movement end to the trial end (2s after the beep cue for the movement initiation). Thus, the force duration was around 1600 ms. We have added the explanation for the duration of the constraint force to the method on lines 620-621 (page 18).

*Line 756: Though I understand it is indirectly described in the text, it may be clearer to also write down the cost function for the flat models for the reader’s benefit.*

Following the reviewer’s suggestion, we have added the equation (Eq. 5) for the cost function of the flat OFC model to the method on lines 768-770 (page 23).

*Reviewer #3: 

First, I felt that the narrative and interpretation from the authors could be made clearer. I suspect that the authors were attempting to remain agnostic or cautious, which may have muddled their narrative. I was left wondering whether they think this effect is truly driven by some form of an explicit re-aiming strategy (an interpretative narrative) or if their intent was simply to emphasize an effect of the presence of target errors (a descriptive narrative). This extends to the discussion of their modeling efforts where it is unclear if they think adaptation to the kinematic plan is the automatic result of target errors or reflects volitional changes (i.e., explicit strategy). Again, I suspect this is caution on the authors part, but I think they need to be “explicit” about it so that the reader can clearly know the authors position and, as such, correctly cite the work.*

Thank you for your suggestion. We believe the kinematic plan adaptation we showed is a sort of explicit strategy learning. To clarify our belief, we have modified some sentences in the discussion on lines 441-443 (page 13). However, we still need further validation to confirm the explicit nature of the kinematic plan. We thus have added this point as a limitation of this study and a future work to the discussion on lines 525-532 (page 15).

*Second, there is a conflation between the physical manipulation and psychological manipulation in the task. We need to be aware that target errors, or task performance errors, are a psychological construction of the participant. Instead of changing the framing of the task, the authors chose to manipulate target errors by the physical properties of the task. In experiment 1, there is a large physical difference between velocity- and position-dependent force field, which would require different control strategies to counter regardless of the psychological evaluation of the participant, yet these are compared as equivalent except for their target error inducing properties. This difference in the physical properties is mitigated by their simulation results, showing that the flat model cannot replicate the learning function of the participants; however, the model when including the directional bias term only qualitatively matches the data. The subtleties that are induced by the physical differences really complicate a convincing, easy-to-see behavioral distinction that could be attributable to a change in task performance criterion/framing.

Their second approach, which is commendable, is to create force field conditions that mix position and velocity perturbations to overcome this issue but here the data here are not convincing either. There’s a numerical (if not significant) bias in de-adaptation in the position-skewed-position dependent field. This could be a spurious finding or it could be power issue. I would also suggest that these experiments be fully presented in the manuscript rather than only partially (with the majority in the supplemental). Their final approach is to use a partial error clamp over the second half of reach to remove target errors. Again, though this requires a change in the physical aspects of the task and, thus, it is conflated with psychological interpretation of target error.*

*I would suggest that the authors take an approach where they simply change the participants’ psychological/conceptual framing of task performance instead of through the perturbations. This could easily be accomplished through instruction, changing the target size, providing points or monetary incentive, task performance criterion etc. This would also help clarify if the directional bias they observe is the result of a control strategies in response to position-dependent field induced target error or if the directional bias arises simply from a volitional change in aiming direction by the subject. In the end, I appreciate the intention of the current study and found the model to be surprisingly useful, but I think a more psychological approach to manipulating task performance could overcome confounds with changing the perturbations.*

Thank you for your suggestions. We appreciate the reviewer’s concern and like the reviewer’s ideas for possible psychological interventions. However, like any study, we are unable to control all the variables between the force fields. We believe both the psychological and physical interventions are coupled, and the overall behavior is a consequence of the interaction between the two, and it is not obvious to us why changing one type would be better than the other- as each has its assumptions and constraints.

But overall, we agree with the reviewer and recognize that there may still be other variables that we cannot control in this study, and that may contribute to our observations. We have therefore acknowledged this point explicitly as a limitation of our study in the discussion on lines 507-523 (page 15). The inability to distinguish the level of volitional (explicit) processing is definitely one of them and the reviewer’s idea of manipulating the target size and reward seems to be a very good way to answer this question and we intend to use it in our follow up study.

In regard to the data reliability for the PSPF and CPVF conditions of Experiment-2 - Please note that the learning curve of LD in Fig S1A is drawn with the raw values but not the baseline-subtracted values unlike Fig S1B. Our statistical analyses reliably suggest that the new null trajectory appears in the CPVF (TE-inducing field) but not the PSPF (non-TE-inducing field). We have checked the effect sizes to confirm that the magnitude of baseline-subtracted TE (131-150 de-adaptation trials) was reliably different from zero in the CPVF (t(13)=3.386, p=0.0049, cohen’s *d*=0.905) but not the PSPF(t(14)=0.629, p=0.540, cohen’s *d*=0.1702) (Figs. 3A, B).

CPVF and PSPF are more of an ‘additional’ supporting experiments for us to examine the possible causes of the new null trajectory observed in the de-adaptation phase. We show the summarized CPVF and PSPF data in the main text but feel that the learning curves over the whole experiment period amounts to too much information for the readers. We therefore prefer to keep it in the supplementary materials unless the reviewer insists otherwise.

*Another two changes*

In addition to the points pointed out by the reviewers, we have changed the following two points regarding Figure 3A.

1) We have redrawn the participant-averaged trajectories as we found some mistakes in the code for this. However, this change is limited to the plotting and does not affect any statistical results and our conclusion which we have reported in the manuscript.
2) The description of how we draw the trajectories was lacking. We have described that in Methods on the lines 679-683 (page 20).
